# Capsule optoacoustic endoscopy for esophageal imaging

**DOI:** 10.1002/jbio.201800439

**Published:** 2019-06-25

**Authors:** Hailong He, Antonios Stylogiannis, Parastoo Afshari, Tobias Wiedemann, Katja Steiger, Andreas Buehler, Christian Zakian, Vasilis Ntziachristos

**Affiliations:** ^1^ Institute of Biological and Medical Imaging Helmholtz Zentrum München Neuherberg Germany; ^2^ Chair of Biological Imaging and TranslaTUM Technische Universität München Munich Germany; ^3^ Institute for Diabetes and Cancer Helmholtz Zentrum München Neuherberg Germany; ^4^ Department of Pathology Klinikum Rechts der Isar, Technical University of Munich Munich Germany

## Abstract

Detection and monitoring of esophageal cancer severity require an imaging technique sensitive enough to detect early pathological changes in the esophagus and capable of analyzing the esophagus over 360 °in a non‐invasive manner. Optoacoustic endoscopy (COE) has been shown to resolve superficial vascular structure of the esophageal lumen in rats and rabbits using catheter‐type probes. Although these systems can work well in small animals, they are unsuitable for larger lumens with thicker walls as required for human esophageal screening, due to their lack of position stability along the full organ circumference, sub‐optimal acoustic coupling and limited signal‐to‐noise ratio (SNR). In this work, we introduce a novel capsule COE system that provides high‐quality 360° images of the entire lumen, specifically designed for typical dimensions of human esophagus. The pill‐shaped encapsulated probe consists of a novel and highly sensitive ultrasound transducer fitted with an integrated miniature pre‐amplifier, which increases SNR of 10 dB by minimizing artifacts during signal transmission compared to the configuration without the preamplifier. The scanner rotates helically around the central axis of the probe to capture three‐dimensional images with uniform quality. We demonstrate for the first time ex vivo volumetric vascular network images to a depth of 2 mm in swine esophageal lining using COE. Vascular information can be resolved within the mucosa and submucosa layers as confirmed by histology of samples stained with hematoxylin and eosin and with antibody against vascular marker CD31. COE creates new opportunities for optoacoustic screening of esophageal cancer in humans.
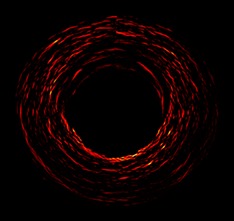

## Graphical abstract

In this work, we introduce a novel capsule optoacoustic endoscopy (COE) system that provides high‐quality 360‐degree images of the entire lumen, specifically designed for typical dimensions of human esophagus. The pill‐shaped encapsulated probe consists of a novel and highly sensitive ultrasound transducer fitted with an integrated miniature pre‐amplifier. We demonstrate for the first time ex vivo volumetric vascular network images to a depth of 2 mm in swine esophageal lining using COE.

## 1 INTRODUCTION

Earlier detection of esophageal cancer can significantly increase the survival rate of humans 1, 2. The stage of esophageal cancer depends on the extent of pathological damage to the esophageal wall and how deeply the damage extends through the multilayer structure of the wall 1, 2, 3. Ultrasound endoscopy is a clinical imaging modality used for esophageal cancer staging and it provides high‐speed and high‐resolution cross‐sectional images over large fields of view 4. However, poor tissue contrast limits the ability of ultrasound endoscopy to identify superficial layer structures, resulting in low accuracy of earlier esophageal cancer staging 5. Recent optical endoscopic imaging modalities have been proposed, such as endoscopic optical coherence tomography (OCT) 6, 7 and confocal endoscopy 8, 9, with promising results for improving disease detection and identifying dysplasia. OCT offers a better outlook for inspecting the entire organ but reveals only morphological contrast at superficial depth 6. Confocal endoscopy allows inspection of only a small field of view at any one time, and it is poorly suited for surveillance of the entire esophageal wall 8, 9.

Optoacoustic endoscopy (COE) avoids these limitations of ultrasound and purely optical endoscopy and so promises to be a superior method for detecting esophageal pathology. Optoacoustics offers advantages over purely optical imaging of tissue because it combines optical contrast with detection of acoustic waves, which are scattered much less than light when passing through tissue. This allows optoacoustics to generate higher resolution images deeper within tissue than conventional optical imaging 10, 11. Previous studies of COE have successfully resolved superficial vascular structures of the gastrointestinal tracts of small animals 12, 13, 14, 15, 16. For example, the esophagus and colon of rats and rabbits have been imaged in vivo using an optoacoustic imaging probe with a diameter of 2.5 mm, which can fit through the working channel of a standard video endoscope 13, 14. Besides, optical‐resolution COE achieved high‐resolution images while providing very limited penetration depth 15, 17, 18.

Although great progress in the field of COE has been achieved in recent years, the miniaturized probes used in those small‐animal studies may not be easy to scale up for use in humans for several reasons. First, the esophageal diameter is much larger in humans than in rats or rabbits, and deeper imaging is required to resolve structures within the wall. This requires appropriate probe geometries to ensure optimal acoustic detection around the 360° circumference. Second, current optoacoustic endoscopic configurations do not provide optimal acoustic coupling and mechanical stability against the entire circumference of the esophageal wall, allowing freedom of motion between the probe and the esophageal wall, which can introduce motion artifacts, preventing 360° volumetric imaging. Lastly, although small endoscopic probes can be inserted into the working channel of commercially available endoscopes, transducers used in such probes have small sensing areas and thus limited sensitivity. Furthermore, optoacoustic signals of endoscopy setups are normally weak and are transmitted through long electrical cables prior to amplification or acquisition, introducing additive noise and interference artifacts, resulting in very low signal‐to‐noise ratio (SNR).

In order to overcome these limitations, we propose a novel capsule COE probe equipped with a highly sensitive transducer and sized to fit snugly in the esophagus of human adults to ensure optimum acoustic coupling for volumetric scanning. A highly sensitive optoacoustic detector is designed using a side‐looking focused ultrasound transducer with an integrated miniature preamplifier circuit to increase SNR. The transducer is implemented with a long working distance to enable imaging of lumen sizes comparable to human esophagus. We describe the COE configuration and present results on full‐circumference imaging with phantoms and excised swine esophagus for the first time. The vascular network observed with the COE images was confirmed by staining the tissue against the endothelial cell marker CD31. This work proposes a new approach for effective translation of optoacoustic techniques to human endoscopic applications.

## 2 METHODS AND MATERIALS

### 2.1 System design

**Figure 1:**
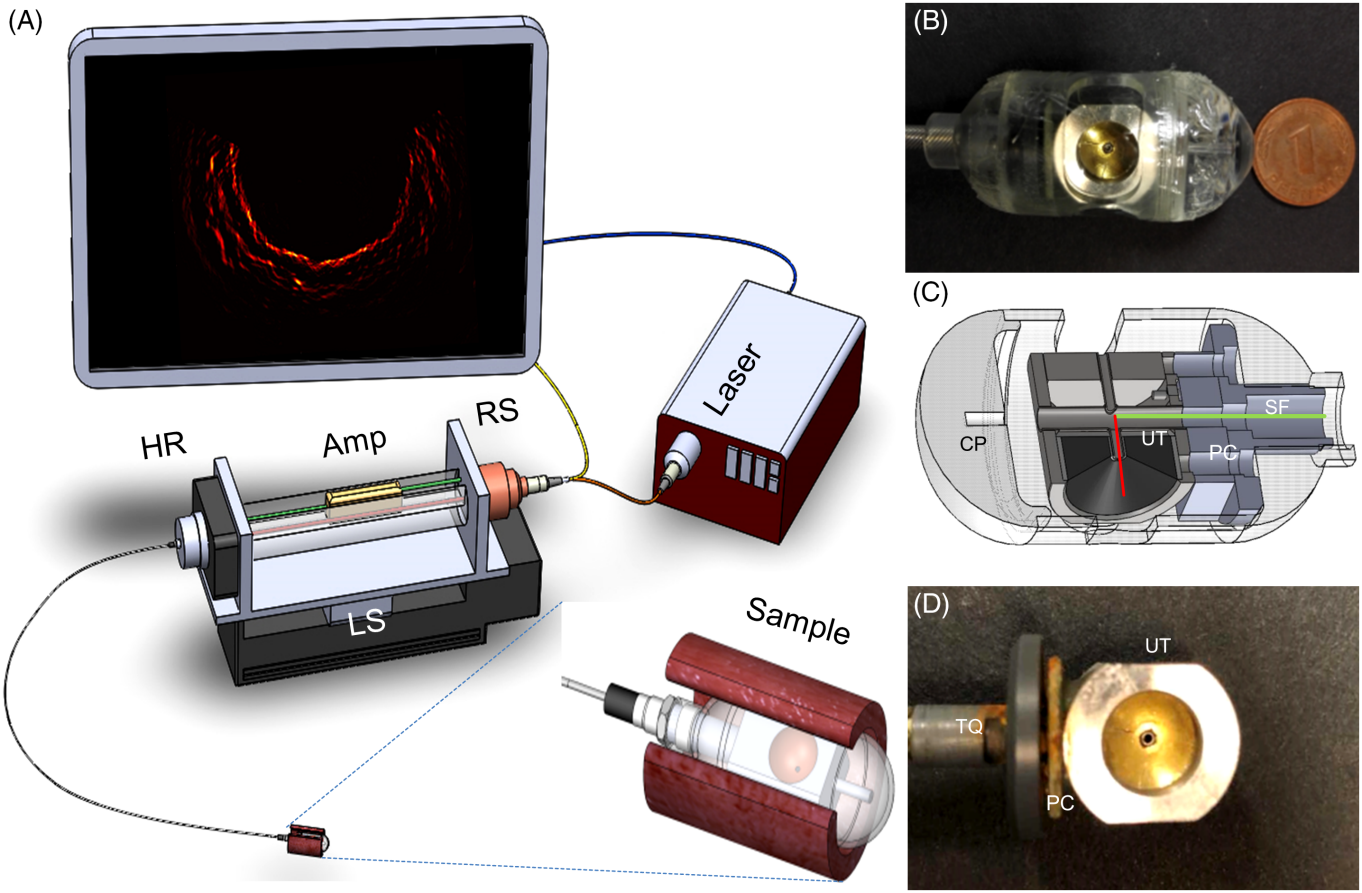
(A) Schematic of the imaging setup. (B) Photograph of the capsule probe. (C) Detailed cut‐away schematic view of the capsule probe. The green line indicates the side view fiber. The red line represents the laser beam. (D) Optoacoustic probe. Amp, amplifier; CP, capsule; HR, hybrid rotary joint; LS, linear stage; PC, preamplifier circuit; RS, rotation stage; SF, side‐looking fiber; TQ, torque coil; UT, ultrasound transducer

The schematic of the endoscopy system and photograph of the distal end of the capsule probe are shown in Figure 1A. A custom‐designed spherical focused PVDF transducer (UT, Precision Acoustics, Dorchester, UK) with a central frequency of 30 MHz and focal length of 7 mm was used for ultrasound detection. The diameter of the transducer was 8 mm, and it had a central aperture with a diameter of 1 mm as shown in Figure 1B,C. For optoacoustic signal generation, we utilized a 532‐nm laser with a pulse repetition rate of 2 kHz, energy of 1 mJ/pulse, and pulse width of 0.9 ns (Wedge HB532, BrightSolutions, Pavia, Italy). The laser beam was coupled into a side‐looking fiber [SF; NA 0.19, core diameter 400 μm, total diameter 800 μm; *green line* in Figure 1C], which was inserted through a central channel with 1 mm diameter in the transducer case, as shown in Figure 1C. The light transmitted through the fiber was carefully aligned to propagate through the central aperture of the transducer, as illustrated by the red line in Figure 1C. This feature enabled coaxial illumination and detection, achieving a working distance of approximately 7 mm, which was determined by the ultrasound focal length. This configuration yielded an optical fluence of 11.6 m J/cm^2^ at the surface of the capsule in contact with the tissue, which is lower than the safety limit defined by the American National Standards Institute. The optoacoustic probe was encapsulated and centered in a custom‐designed capsule pill with diameter of 18.6 mm and length of 20 mm, as shown in Figure 1B. The focus of the ultrasound transducer was positioned at the outer surface of the capsule. For better optical and acoustic propagation, three identical windows 10 mm long were created on the cylinder surface of the capsule as shown in Figure 1B and were covered with a transparent membrane (polyvinyl chloride, 25 μm thick).

In optoacoustic endoscopy, the employed ultrasound transducers usually have limited size and very low series capacitance, which results in impedance mismatch in the signal transmission from the transducer to the co‐axial cable. Besides, the weak optoacoustic signal has to travel a long distance to the external amplifier through the co‐axial cable, easily distorted by external interference artifacts and additive noise. Furthermore, electrical rotation coupling can distort the optoacoustic signal, resulting in low SNR. In order to overcome this issue, a miniature preamplifier circuit (PC; 30 dB amplification, ERA‐8SM+, Mini‐Circuits, Brooklyn, New York) was designed and mounted directly on the ultrasound transducer case before the signal was transmitted to the console, as shown in Figure 1D. In this arrangement, we achieve an impedance matched transmission of the amplified signal that is less affected by the long transmission distance and electrical rotation coupling.

The ultrasound signal was transmitted through a coaxial electrical cable 1.5 m long and coupled to a hybrid rotary joint (HR; Princetel, Hamilton, New Jersey), amplified by a low noise amplifier (Amp; 30 dB, Miteq, Hauppauge, New York) and recorded by a high‐speed digitizer operating at 200 MS/s (PCI‐5124, National Instruments, Austin, Texas; 12‐bit resolution; max sampling rate 4 GS/s). The electrical transducer cable (1.5 m long) and optical fiber were inserted inside a hollow custom‐designed torque coil (TC: Asahi, Japan) with outer diameter of 3.2 mm. This coil accurately transferred the rotation scanning force to the endoscopy probe and not the capsule. Fast linear and rotation stages (LS: EZS‐AR, RS: DG60; Oriental Motor, Tokyo, Japan) were combined to produce a 3D helical scanning pattern.

### 2.2 Sample preparation

**Figure 2:**
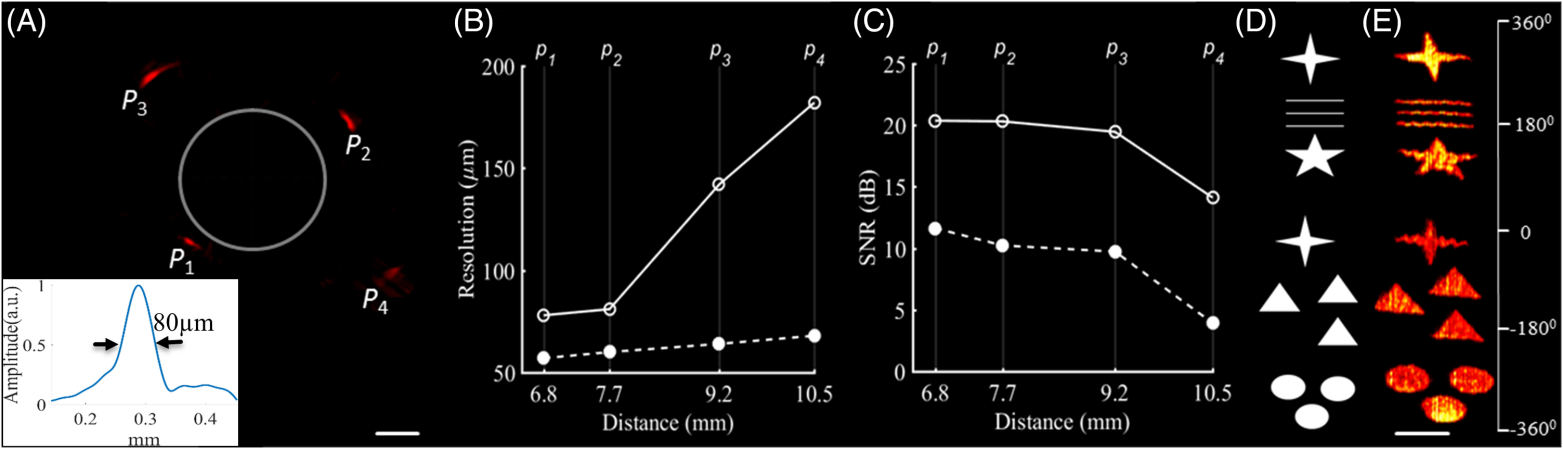
Phantom imaging with the COE probe. (A) Optoacoustic image of the phantom obtained with the probe (outlined as a solid gray circle), in which four sutures (labeled as *p*_1_*p*_4_) were positioned around the capsule at different distances. The inset is the point spread function of point*p*_1_. Scale bar, 2 mm. (B) Transversal (solid line, open circles) and radial resolutions (dotted line, solid circles) along the depth direction, calculated from the FWHM values of the four sutures. (C) SNR for each suture in the phantom obtained following optoacoustic imaging with the miniature preamplifier attached to the transducer (solid line, open circles) or without the preamplifier (dotted line, solid circles). (D) Schematic of the geometric shapes printed on paper, and rolled around the capsule probe. (E) Corresponding RMIP (radial maximum intensity projection) image of the paper phantom in panel (E). Scale bar, 2 mm

A phantom consisting of four sutures (10 μm in diameter) was made to characterize the performance of the developed probe. Sutures were fixed around a hollow cylinder at different radial distances from the center. The capsule was placed inside the cylinder to mimic the scanning geometry of a luminal organ as shown in Figure 2A. Radial and transverse resolutions were quantified by calculating full width at half‐maximum (FWHM) values of the suture image. The effect of the miniature preamplifier on SNR was evaluated by measuring the sutures in the same configuration with and without the circuit attached to the ultrasound transducer. The SNR was calculated as 10 × log_10_(*I*_max_/*I*_b_), where *I*_max_ is the maximum intensity and *I*_b_ is the SD of the image background. The volumetric imaging performance was tested using a 6‐mm‐long white paper with simple geometric shapes printed on the surface, which was rolled into a cylinder around the capsule as shown in Figure 2E. Both the suture and paper phantom were immersed in water for better acoustic coupling.

In addition, a fresh ex vivo sample of swine esophagus was imaged to validate the feasibility of the proposed imaging approach; swine esophagus is similar in histology and dimensions to human esophagus. Animal experiments were approved by the Government of Upper Bavaria (permit number 55.2‐1‐54‐2532‐6‐13) and performed according to the German Animal Welfare Act and European Union Normative for Care and Use of Experimental Animals. A 1‐year‐old male pig was sacrificed and the esophagus was immediately resected. The sample was placed on ice with the ends tightened to ensure that the blood would remain in esophageal vasculature and coagulate naturally. A 10‐cm section of the middle esophagus was carefully dissected to preserve the layer structure, including the mucosa, submucosa and supporting muscle. Acoustic gel was applied to the external part of the capsule pill and inserted through the lumen of the esophagus sample, ensuring good contact against the inner mucosal wall. The capsule was filled with deionized water for acoustic coupling.

### 2.3 Imaging protocol

For the phantom and esophagus measurements, each A‐scan was captured following the trigger from a laser pulsing at a repetition rate of 2 kHz. A laser wavelength of 532 nm was chosen because it is close to an isosbestic point in the absorption spectrum of hemoglobin, which would allow us to map the vascular morphology of the sample independent from blood oxygenation. The probe was helically scanned at a rotation speed of 2.5 Hz and a pitch of 10 μm for three‐dimensional imaging by translating the probe inside the capsule. A total of 800 A‐line signals were recorded per rotation without averaging. Each A‐scan consisted of 2000 points with depth information; before image reconstruction, the first 600 time points were removed as they contained depth information corresponding to the initial ultrasound wave traversing the capsule prior to the mucosal surface signal. The recorded signals were bandpass‐filtered between 10 and 50 MHz and Hilbert‐transformed for visualization.

### 2.4 Histology

Immediately after imaging, the ex vivo sample was cut along the lumen to open the esophagus flat and allow dissection of the area of interest. The samples were fixed with 4% paraformaldehyde for at least 24 h and embedded in paraffin. Paraffin‐embedded tissues were cut into 4‐μm sections, which were stained with hematoxylin and eosin and examined by light microscopy. Images were recorded on an Olympus BX43 microscope equipped with an Olympus DP25 camera and Olympus LabSense software.

### 2.5 CD31 Immunohistochemistry

Immunohistochemistry was performed on the ex vivo sample using the Bond RXm system (Leica, Wetzlar, Germany) and primary antibody against CD31 (ab28464; Abcam, Cambridge, UK) diluted 1:50 in Bond Primary Antibody Diluent (AR9352, Leica). Slides were deparaffinized using deparaffinization solution, and treated for 30 min with Epitope Retrieval Solution 2. Antibody binding was detected with a Bond Polymer Refine Detection kit (DS9800, Leica) without post‐primary reagent and visualized with DAB as a dark brown precipitate. Immunostained sections were counterstained with hematoxylin.

## 3 RESULTS

After developing the COE probe, we tested its performance on a suture phantom (Figure 2). Figure 2A shows a cross‐sectional optoacoustic image. The radial and transverse resolutions were quantified by calculating FWHM values from the corresponding optoacoustic intensity line profiles across the sutures. The highest spatial resolution was obtained at the focal distance of the transducer (about 6.8 mm), yielding a transverse resolution of 80 μm and a radial resolution of 55 μm. Figure 2B shows that, as expected, the transverse resolution dropped as the radial distance increased, giving an imaging distance of about 1 mm, which corresponded to the length of the focal zone of the transducer. The radial resolution is determined primarily by the bandwidth of the transducer, and therefore it varied to a smaller extent in this direction. We performed two sets of experiments, one without the preamplifier and one with the preamplifier connected to the UST. The results are presented in Figure 2C and show that integrating the miniature preamplifier into the system increased SNR by approximately 10 dB for all four sutures. To test the probe's ability to image various structures, we printed geometric shapes on white paper (Figure 2D). Figure 2E shows the corresponding radial maximum intensity projection (RMIP) optoacoustic image. The absorbing structures were recovered with uniform image quality, demonstrating the volumetric imaging capability of the system. The edges of some objects displayed slight distortions because of printer inaccuracy.

**Figure 3:**
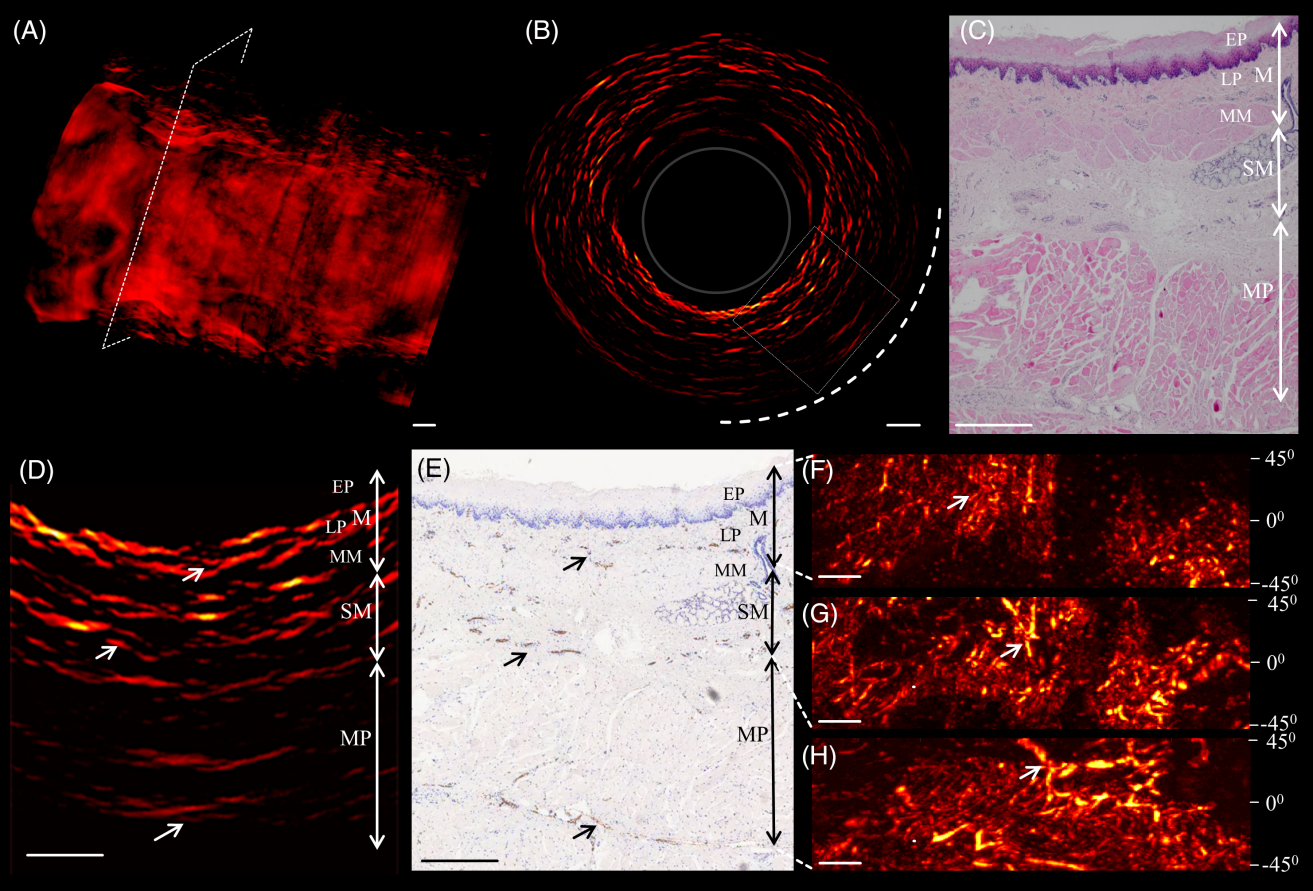
Imaging of pig esophagus ex vivo with a COE probe. (A) Volumetric image of the esophagus sample. (B) Full 360° cross‐sectional optoacoustic image of the region of esophageal wall boxed in panel (A). The image is a radial maximum intensity projection over a 50‐μm slab. The dashed box delineates the region shown as a zoomed‐in area in panel (D). (C) Histological image from the section with the different layers of the esophagus wall indicated. COE, optoacoustic endoscopy; EP, epithelium (sublayer of mucosa); M, mucosa; LP, lamina propria (sublayer of mucosa); MM, muscularis mucosa (sublayer of mucosa); MP, muscularis propria; SM, submucosa. (D) Zoomed‐in image of the boxed area in panel (B). (E) Vasculature of the different esophageal layers revealed by anti‐CD31 immunostaining. (F‐H) RMIP images of, respectively, the M, SM and MP layers of the esophageal wall. Arrows in panels (D)‐(F) indicate vessel structures. All scale bars, 500 μm

Encouraged by these results, we continued using the preamplifier and tested the COE probe on ex vivo sections of pig esophagus as shown in Figure 3. Figure 3A shows a volumetric optoacoustic image (1 cm long) of the esophagus sample, while Figure 3B shows a representative 360° cross‐sectional RMIP of the esophagus wall over a 50‐μm slab close to the plane marked by the dashed line in Figure 3A. The region enclosed by the rectangle in Figure 3B is shown as a zoomed‐in image in Figure 3D, and the corresponding histology sections are shown in Figure 3E after staining with hematoxylin and eosin and in Figure 3E after anti‐CD31 immunostaining. The histology section in Figure 3C illustrates the layered morphology of the esophageal wall, which includes the mucosa (M), which comprises the epithelium (EP), lamina propria, and muscularis mucosa (MM); the submucosa (SM); and the muscularis propria (MP) 19. The optoacoustic sectional image in Figure 3D highlights the vascular network within the esophagus layers. The arrows in Figure 3D,E and highlight vasculature features in different esophagus layers, which correlated well with positive immunostaining for the endothelial marker CD31. This comparison suggests that the vascular morphology of the esophagus wall can be resolved as deep as 2 mm below the surface. As expected, vascular structures were not observed in the EP layer, reflecting the fact that the vascular network lies within and deeper than the LP layer. The dark areas corresponding to the MM layer in Figure 3D likely contain glands, muscle fibers and connective tissue, which are expected to generate weaker optoacoustic signal than hemoglobin after illumination at 532 nm. Interestingly, vasculature appeared less dense within the MP, which correlated with weaker anti‐CD31 immunostaining. Signals closer to the lumen surface were generally stronger, reflecting the fact that light fluence decreases as light penetrates deeper in tissue. Figure 3F‐H show RMIPs of the volumetric region along the longitudinal direction of the esophagus within the dashed quarter‐circle in Figure 3B; these RMIPs belong, respectively, to the M, SM and MP layers. The resolution of COE was sufficient to observe the expected increase in vessel diameter with increasing depth within the esophageal wall, as indicated by the white arrows in Figure 3F‐H. In this way, assessment of vessel diameter in COE may be useful for differentiating layers in the esophageal wall.

## 4 DISCUSSION

We show for the first time volumetric imaging of large‐animal esophagus using a novel COE system optimized for human use. The average lumen diameter of pig esophagus is 25 mm, far larger than the diameter of 3 to 5 mm in previous COE studies in rodents and rabbits, and comparable to the average lumen diameter of human esophagus (20 mm). The COE probe resolved vascular structure and different layers of the esophageal wall down to a depth of 2 mm. To the best of our knowledge this is the first demonstration of deep optoacoustic imaging in swine esophagus tissue, which was facilitated by integrating a high‐sensitivity transducer and a miniature preamplifier, as well as by designing device dimensions that ensure good acoustic coupling over the 360° circumference. This work establishes the feasibility of high‐resolution COE for larger animals and humans.

Previous COE implementations have been limited to imaging small lumens with diameters of 2 to 3 mm in rats and rabbits 13, 14. For instance, one set‐up was reported to offer a working distance of 4 mm with a scanning circumference of 270 to 310° 13 . Another set‐up was reported to allow a 360° field of view for imaging the colorectum of rats 14. These set‐ups can be potentially integrated into the working channel of commercially available endoscopes, but their small working distance and limited field of view make them ill‐suited for human use. The limited working distance poses acoustic coupling and SNR challenges in large esophageal lumens. In addition, using these probes within the working channel of clinical endoscopes often means the probes are off‐center, causing the distance between probe and tissue to vary during the circumferential scanning. Moreover, optical resolution‐based COE probe achieved high resolution while very limited penetration depth.

Capsule endoscopes allow for stable esophagus lumen distension to a desired diameter, as previously explored for OCT 7. However, the feasibility of this approach has not yet been demonstrated for optoacoustic imaging. The COE is designed with an outer diameter of 18 mm, providing sufficient space to host a customized, highly sensitive, focused ultrasound transducer with a novel miniature preamplifier that increases the SNR by 10 dB, as shown in Figure 2.

This preamplifier enabled to achieve better impedance match between the transducer and the co‐axial transmission cable, which significantly helped to minimize the negative impacts of noise and artifacts, and electrical rotation coupling interference that arise during transmission through the long cable.

The transducer operates at a working distance of 7 mm and the capsule is rotated and pulled back with a torque coil to obtain uninterrupted, full‐view, 360° scans along the esophagus length.

The ability to resolve the superficial layer structure of the esophagus wall is crucial for detecting and staging esophageal cancer as early as possible. Esophageal cancer stage depends mainly on the depth of the infiltration and the extent of damage to histological layers of the esophagus wall. Our images suggest that COE based on the endogenous optical contrast of hemoglobin can differentiate the superficial layer structures of the esophageal wall, which should be helpful for detection of early esophageal cancer. In addition, it may be possible to implement multi‐wavelength capability into the COE probe. This may allow the visualization of functional information, such as the oxygen saturation of blood, or molecular information, such as the distribution of various contrast agents or molecular probes. Such a rich range of information may help to increase the sensitivity and reliability of COE as a diagnostic tool, as well as increase the amount of information that can be gained about a patient's condition without the need for invasive biopsy.

We show for the first time the ability to volumetrically image entire swine esophagus. Vascular structure and layer features of the esophagus wall were resolved in the mucosal and submucosal layers to an imaging depth of 2 mm. We demonstrate that COE can map the vascular network within the different layers of excised esophageal tissue, and we confirmed this using anti‐CD31 immunostaining. At the same time, the ability of the COE to differentiate smaller capillaries through the esophageal layers and sublayers within the mucosa is limited by the fact that signal strength and limited axial resolution. It may be possible to improve the resolution by using ultra‐wide bandwidth transducers ranging from a few MHz up to hundreds of MHz, based on our previous work 20. Another area for improvement in the COE design are the three windows on the capsule pill, which were covered with a membrane allowing high acoustic and optical transmission. It may be possible to avoid the need to make windows by using novel materials transparent to sound and light. Furthermore, micro motors for rotation scanning can be integrated inside the capsule probe, which can increase the scanning accuracy for in vivo measurements compared to external rotation mechanism. For current setup, the scanning speed is 2.5 Hz, which is mainly limited by the repetition rate of laser source. Fast laser source will be applied for in vivo measurements in the future. Beside optoacoustic imaging, other imaging modalities, for example OCT/ Doppler OCT can be integrated inside the capsule probe. The dual‐modality system can provide complimentary information: fast high resolution OCT images of the superficial structures and deeper penetration depth of optoacoustic images 21. Lastly, a mini analog to digital converter could be integrated on the preamplifier PCB which could further increase the SNR and reduce transmission loss.

Overall, the results from this study suggest a new implementation of optoacoustics suitable for screening large gastrointestinal tracts with promising opportunities for translation to human esophageal imaging.
